# Commentary: Cognitive reflection vs. calculation in decision making

**DOI:** 10.3389/fpsyg.2016.00009

**Published:** 2016-01-22

**Authors:** Gordon Pennycook, Robert M. Ross

**Affiliations:** ^1^Department of Psychology, University of WaterlooWaterloo, ON, Canada; ^2^Department of Psychology, Royal Holloway, University of LondonLondon, UK; ^3^ARC Centre of Excellence in Cognition and its Disorders, Macquarie UniversitySydney, NSW, Australia

**Keywords:** numeracy, Cognitive Reflection Test, biases, individual differences, dual-process theory

Sinayev and Peters (2015; hereafter S&P) present two competing hypotheses to explain performance on the Cognitive Reflection Test (CRT). They dub the first the “Cognitive Reflection Hypothesis” and attribute it to other researchers: “Each of these researchers assumes that differences in CRT performance indicated differences in the ability to detect and correct incorrect intuitions… ” and “… implicitly assume that numerical ability is an irrelevant detail when it comes to solving CRT and related problems” (p. 2). They contrast this with their “Numeracy Hypothesis” which states that “the CRT is primarily a measure of numeric ability” (p. 3). S&P report two studies whose results, they argue, favor the Numeracy Hypothesis over the Cognitive Reflection Hypothesis. They conclude that numeric ability is “the key mechanism” that explains the association between CRT performance and decision making (p. 1), although they also state that the ability to detect and correction intuitions (apart from numeracy) plays a role in CRT performance. Both of the hypotheses presented by S&P emphasize the role of cognitive ability in CRT performance. In this commentary we introduce an alternative hypothesis that was not discussed by S&P; namely, that the *propensity* or *disposition* to think analytically plays an important role in CRT performance (Pennycook et al., 2015b). We discuss recent empirical evidence that supports the claim that the CRT is more than just a measure of numeracy or, more generally, cognitive ability.

## Distinguishing cognitive ability and analytic cognitive style

Dual process theorists often distinguish between *disposition* and *ability* as factors that determine good reasoning (e.g., Stanovich and West, 2000; Stanovich, 2009; Evans and Stanovich, 2013). The logic is as follows: If someone does not have the disposition or willingness to think analytically, they will not fully exercise their cognitive ability and will not do as well on the problem. Naturally, the converse is also true: If someone does not have sufficient cognitive ability, it will not matter how much time and effort they are willing to spend thinking about the problem.

This distinction has been applied to CRT performance. For example, according to Toplak et al. (2014): “the CRT is a measure of the tendency toward the class of reasoning error that derives from miserly processing. This may be why the predictive power of the CRT is in part separable from cognitive ability. The latter measures computational power that is available to the individual, but not necessarily the depth of processing that is typically used in most situations” (p. 165). That each question in the CRT cues a compelling intuitive response means that responding correctly requires that individuals expend cognitive effort despite having what initially appears to be a suitable response (Pennycook et al., 2015b). Importantly, this focus on thinking disposition does *not* imply that cognitive abilities (such as numeracy) are irrelevant for CRT performance. Rather, the claim is that the CRT indexes, to some degree, a *disposition* or *propensity* to think analytically (i.e., “analytic cognitive style”) *in addition to* cognitive ability. Prima facie evidence for the importance of thinking disposition in solving the CRT comes from the finding that few people provide the correct responses (e.g., 30.3% for the bat and ball problem among undergraduate students; Pennycook et al., 2015b) despite the apparent simplicity of the math required to check the accuracy of the intuitive response (e.g., for the bat and ball problem: 0.10+1.10 = 1.20 ≠ 1.10).

## Is the CRT just another numeracy test?

If the CRT captures some aspect of analytic cognitive style, it should be predictive of a wide range of judgments and decisions. However, if the CRT is “primarily a measure of numeric ability” (S&P, p. 3), then it should only robustly predict judgments and decisions that require some sort of mathematical operation.

There is emerging evidence that analytic cognitive style—and the CRT in particular—is predictive of diverse psychological outcomes that are not traditionally associated with research in decision making (Pennycook et al., 2015c). For instance, higher scores on the CRT are associated with religious disbelief (Gervais and Norenzayan, 2012; Pennycook et al., 2012; Shenhav et al., 2012), paranormal disbelief (Pennycook et al., 2012; Cheyne and Pennycook, 2013), less traditional moral values (Pennycook et al., 2014; Royzman et al., 2014), improved scientific understanding and reasoning (Shtulman and McCallum, 2014; Drummond and Fischhoff, 2015), belief in evolution (Gervais, 2015), creativity on complex tasks (Barr et al., 2015a), less reliance on Smartphone technology as an external information source (Barr et al., 2015b), and lowered receptivity to pseudo-profound bullshit (Pennycook et al., 2015a). Indexes of cognitive ability were included as controls in many of these studies (Pennycook et al., 2015c), including, in some cases, established numeracy tests (Pennycook et al., 2014, 2015a; Barr et al., 2015a,b; Trippas et al., 2015). With few exceptions, analytic cognitive style measures (including the CRT) were predictive after controlling for cognitive ability (including numeracy; Pennycook et al., 2015c).

What, then, of the two new studies presented by S&P? That CRT performance was not predictive over-and-above numeracy may simply provide evidence that the aspect of CRT performance that reflects thinking disposition does not play a role in the types of decisions that S&P investigated. This seems particularly likely with respect to the incentivized outcomes in Study 2 (as discussed by S&P, p. 12) because the very goal of incentivizing tasks in behavioral research is to minimize dispositional variance. We suggest that a stronger test of the role of thinking disposition over-and-above numeracy would be in judgments or decisions in “naturalistic” contexts where there is no clear prompt or direct incentive to think analytically (Stanovich et al., 2013). S&P did measure some real-world outcomes (e.g., saving money for retirement). However, examined outcomes all included direct incentives (e.g., monetary reward). The evidence highlighted above indicates that CRT performance is predictive for beliefs or judgments that not only lack incentives, but lack normatively correct or incorrect outcomes.

As a case study, consider the results of Pennycook et al. (2014) presented in Table 1. This study focused on predicting religious belief, traditional moral values (e.g., trust in authority, concerns over bodily purity), and disgust-based moral dilemmas. Importantly, none of these constructs has any theoretical association with numeracy, but they do involve compelling intuitions or societal defaults that could be influenced by the disposition to think analytically (for further detail, see Pennycook et al., 2014). As expected given our account, numeracy and “calculation” (using S&P's CRT scoring technique) are not significant predictors of any outcome variable, whereas “cognitive reflection” is a robust predictor for all three (see Supplementary Materials for further details about this analysis). Note, however, that it would be inappropriate to conclude on this basis that numeracy has nothing to do with CRT performance. Rather, the purpose of this analysis is to show that there are some instances where the CRT predicts an outcome *even after controlling for numeracy*. This indicates that the CRT is more than just a numeracy measure. Similar analyses have been done with a variety of outcome variables and with a variety of cognitive abilities as control variables (see Pennycook et al., 2015c).

**Table 1:** **Re-analysis of Pennycook et al. (2014)**.

**Dependant variable**	**Step**	**Age**	**Gender**	**Education**	**Income**	**Cognitive Reflection**	**Calculation**	**Numeracy**
Religious Belief	Step 1	**0.35**	**0.15**	−0.09	−0.03			
	Step 2	**0.34**	**0.13**	−0.08	−0.03	−**0.19**		
	Step 3	**0.34**	**0.13**	−0.07	−0.03	−**0.19**	0.02	
	Step 4	**0.33**	**0.12**	−0.07	−0.02	−**0.18**	0.03	−0.07
Traditional moral values	Step 1	**0.14**	<0.01	−**0.14**	0.06			
	Step 2	**0.14**	−0.02	−**0.12**	0.07	−**0.20**		
	Step 3	**0.13**	−0.01	−**0.11**	0.07	−**0.21**	0.04	
	Step 4	**0.13**	−0.02	−**0.11**	0.07	−**0.20**	0.05	−0.07
Disgust−based moral judgments	Step 1	**0.13**	**0.18**	−**0.15**	**0.15**			
	Step 2	**0.13**	**0.14**	−**0.12**	**0.16**	−**0.25**		
	Step 3	**0.13**	**0.14**	−**0.12**	**0.16**	−**0.25**	−0.03	
	Step 4	**0.12**	**0.13**	−**0.11**	**0.17**	−**0.23**	−0.02	−0.08

*Stepwise regression results predicting religious belief, traditional moral values, and disgust-based moral judgments. In each case, Cognitive Reflection remains a statistically significant predictor after controlling for Calculation and Numeracy (Step 4). Cognitive Reflection = Proportion of correct responses that were not intuitive on the Cognitive Reflection Test (CRT; Frederick, 2005). Calculation = Proportion of non-intuitive CRT responses that were correct. Numeracy = Proportion correct on Schwartz et al. (1997) three-item test. See Supplementary Materials for further information on this study. Standardized beta coefficients. Gender: 1 = Male, 2 = Female. Significant independent predictors in bold (p < 0.05). N = 378*.

## Conclusion

What is the role of analytic cognitive style and cognitive ability in decision making? Although, the answer undoubtedly depends on the sort of decision being made, we have drawn attention to evidence that the CRT is predictive of a wide range of outcomes, even after controlling for cognitive ability (Pennycook et al., 2015c). This provides evidence that CRT performance reflects, at least to some degree, a propensity or willingness to think analytically and the CRT, therefore, is not “primarily a measure of numeric ability” (S&P, p. 3).

Nevertheless, we acknowledge that a propensity to think analytically does not play an important role in all (or perhaps even most) decisions that people make in their day-to-day lives. Moreover, it is clear that the role of numeracy in CRT performance has not been adequately acknowledged by dual-process theorists. Future research could profitably follow S&P's lead by further investigating which types of decisions depend on numeracy, but not cognitive style (and vice versa). This will require indices of *both* analytic cognitive style and cognitive ability (and, in particular, numeracy), as well as a more nuanced hypotheses about what factors explain performance on reasoning and decision making tasks.

## Author contributions

GP wrote the initial draft of this manuscript. RR provided critical revisions.

### Conflict of interest statement

The authors declare that the research was conducted in the absence of any commercial or financial relationships that could be construed as a potential conflict of interest.
