# Supplementary material for: Commentary: Cognitive reflection vs. calculation in decision making
Source: Front Psychol. 2016 Jan 22;7:9. doi: 10.3389/fpsyg.2016.00009 (PMC4722428; doi:10.3389/fpsyg.2016.00009)
Supplement: Supplementary file 1 [file DataSheet1.docx]

Supplementary Material

Commentary on Cognitive reflection vs. calculation in decision making

Gordon Pennycook*, Robert M. Ross

*** Correspondence:** Gordon Pennycook: gpennyco@uwaterloo.ca

# Supplementary Methods

All quotations are from Pennycook, Cheyne, Barr, Koehler, & Fugelsang (2014, *Thinking & Reasoning*).

**Participants**

“Five hundred and seventy participants were recruited through Mechanical Turk™. Twenty-one participants were excluded because they failed an attention check question presented half way through the procedure. Forty-four were dropped from the sample due to missing data leaving 505 participants (241 females; average age ¼ 30.96, SD ¼ 11.42). Participation was voluntary and participants received remuneration. Following the study, all participants gave permission for their data to be used. Only participants who indicated that they lived in the United States were permitted to do the study2. Sessions lasted approximately 30 minutes.” (*p*. 194-195)

**Materials**

“*Moral judgements*. Participants were given two vignettes describing a disgusting act that is generally viewed as morally wrong (Haidt, Bjorklund, & Murphy, 2000) and asked to rate how morally wrong each of the scenarios were on a 7-point scale from “1—Not morally wrong at all” to “7—Extremely morally wrong” (*p*. 198). We used the following vignettes: 1) “A man goes to the supermarket once a week and buys a dead chicken. But before cooking the chicken, he has sexual intercourse with it. He then cooks it and eats it in the privacy of his own home.” and 2) Julie and Mark are brother and sister. They are traveling together in France on summer vacation from college. One night they are staying alone in a cabin near the beach. They decide that it would be interesting and fun if they tried making love. At the very least it would be a new experience for each of them. Julie was already taking birth control pills, but Mark uses a condom too, just to be safe. They both enjoy making love, but they decide not to do it again. They keep that night as a special secret, which makes them feel even closer to each other. What do you think about that? Was it OK for them to make love?

“These two vignettes were chosen because (a) they were designed to specifically exclude any care- or fairness-based violations of moral values, (b) they elicit particularly strong disgust-based intuitive responses, and (c) they elicit emotionally driven responses that are resistant to reasoned persuasion (Haidt, 2001). Thus, presumably, the incest and zoophilia vignettes cue responses that are particularly resistant to modification via analytic reasoning and therefore provide a strong test of our hypothesis” (*p*. 198).

*Moral Values/Foundations.* “Previous work has established the importance of moral values/foundations in morality (e.g., Graham, et al., 2011; Haidt & Graham, 2007). [We included] a questionnaire aimed at ascertaining participants’ explicit endorsement of various moral principles. Specifically, participants were asked to rate how important 6 *individualizing* and 4 *binding* (traditional) principles were to their moral thinking on a 7 point scale from “1 - Irrelevant” to “7 - Extremely Important” (Graham, et al., 2011). Individualizing values included being kind, supporting the autonomy of others, being helpful, being fair, avoiding harm, and supporting the rights of others. Binding/traditional values included showing respect for traditions, being patriotic and loyal, showing respect for legitimate authority, and being pure by avoiding carnal pleasures and disgusting things. Items were summed for the binding and individualizing subscales for analysis” (p. 198). Only the binding/traditional values are reported in the commentary as individualizing values did not relate to any cognitive measure (i.e., neither CRT nor numeracy).

*Religious belief*. “The religious belief (R_b_; Pennycook, Cheyne, Seli, Koehler, & Fugelsang, 2012) scale assessed nine conventional religious beliefs widely held by religious people: afterlife, heaven, hell, miracles, angels, demons, the soul, Satan, and the effectiveness of prayer. Participants responded on a 6 point scale from “1 - Strongly Disagree” to “6 - Strongly Agree”. (*p*. 198-199).

*CRT*. Participants also answered the three-item Cognitive Reflection Test (see Sinayev & Peters, 2015).

# Supplementary References

Graham, J., Nosek, B. A., Haidt, J., Iyer, R., Koleva, S., & Ditto, P. H. (2011). Mapping the moral domain. *Journal of Personality and Social Psychology, 2*, 366-385.

Haidt, J. (2001). The emotional dog and its rational tail: A social intuitionist approach to moral judgement. *Psychological Review, 108*, 814–834.

Haidt, J., Bjorklund, F., & Murphy, S. (2000). *Moral dumbfounding: When intuition finds no reason.* Unpublished manuscript, University of Virginia.

Pennycook, G., Cheyne, J.A., Barr, N., Koehler, D.J. & Fugelsang, J.A. (2014). The role of analytic thinking in moral judgements and values. *Thinking & Reasoning, 20*, 188-214.

Pennycook, G., Cheyne, J.A., Seli, P., Koehler, D.J. & Fugelsang, J.A. (2012). Analytic cognitive style predicts religious and paranormal belief. *Cognition, 123*, 335-346.

Sinayev, A., & Peters, E. (2015). Cognitive reflection vs. calculation in decision making. *Frontiers in Psychology, 6*, 532.
